# Interleukin-17: A Putative Novel Pharmacological Target for Pathological Pain

**DOI:** 10.2174/1570159X21666230811142713

**Published:** 2023-08-15

**Authors:** Shao-Jie Gao, Lin Liu, Dan-Yang Li, Dai-Qiang Liu, Long-Qing Zhang, Jia-Yi Wu, Fan-He Song, Ya-Qun Zhou, Wei Mei

**Affiliations:** 1 Department of Anesthesiology, Hubei Key Laboratory of Geriatric Anesthesia and Perioperative Brain Health, and Wuhan Clinical Research Center for Geriatric Anesthesia, Tongji Hospital, Tongji Medical College, Huazhong University of Science and Technology, Wuhan, 430030, China

**Keywords:** Interleukin-17, bone cancer pain, neuropathic pain, inflammatory pain, peripheral mechanisms, central mechanisms

## Abstract

Pathological pain imposes a huge burden on the economy and the lives of patients. At present, drugs used for the treatment of pathological pain have only modest efficacy and are also plagued by adverse effects and risk for misuse and abuse. Therefore, understanding the mechanisms of pathological pain is essential for the development of novel analgesics. Several lines of evidence indicate that interleukin-17 (IL-17) is upregulated in rodent models of pathological pain in the periphery and central nervous system. Besides, the administration of IL-17 antibody alleviated pathological pain. Moreover, IL-17 administration led to mechanical allodynia which was alleviated by the IL-17 antibody. In this review, we summarized and discussed the therapeutic potential of targeting IL-17 for pathological pain. The upregulation of IL-17 promoted the development of pathological pain by promoting neuroinflammation, enhancing the excitability of dorsal root ganglion neurons, and promoting the communication of glial cells and neurons in the spinal cord. In general, the existing research shows that IL-17 is an attractive therapeutic target for pathologic pain, but the underlying mechanisms still need to be investigated.

## 1 INTRODUCTION

Pathological pain has an estimated prevalence of 20% in the general population and is a tremendous burden to the economy and the patient’s quality of life [1]. Pathological pain is characterized by allodynia, hyperalgesia, and spontaneous pain [2, 3]. Opioids, the powerful analgesics used by clinicians, have severe side effects, including respiratory depression, tolerance, constipation, addiction, nausea, and vomiting when used for a long time [4]. Therefore, understanding the mechanisms of pathological pain is essential for the development of effective analgesics. Recently, ample evidence indicates that IL-17 may play an important role in the development and maintenance of pathological pain.

IL-17 is a proinflammatory cytokine and is involved in the development of several chronic inflammatory diseases and autoimmune diseases [5-7]. IL-17 mainly originated T helper 17 (Th17) cells, and also originated neutrophils, mast cells, and natural killer (NK) cells [8-10]. The production of IL-17 is primarily associated with the differentiation and proliferation of Th17 cells, and this process is regulated by some cytokines. IL-6, IL-1β, and transforming growth factor β (TGF-β) promote the differentiation of Th17 cells [11, 12]. IL-23 interacts with IL-23R expressed by the differentiated Th17 cells and promotes the proliferation of Th17 cells. Conversely, IL-27 inhibits the production of IL-17 by inhibiting the differentiation of Th17 cells [13]. IL-17 receptor (IL-17R) family includes five receptors: IL-17RA, IL-17RB, IL-17RC, IL-17RD, and IL-17RE, and IL-17RA may be a common receptor chain for IL-17 [14-18]. The combination of IL-17 and IL-17RA can activate the nuclear factor-κB (NF-κB) pathway, and also activate CCAAT/enhancer binding proteins (C/EBPs) C/EBPβ and C/EBPδ to promote several genes transcription, such as inflammatory cytokines, chemokines, and matrix metalloproteinases [19, 20]. Although IL-17 is a proinflammatory cytokine, it is currently thought that its proinflammatory effect is mainly through recruiting immune cells and interacting with other cytokines [21].

Growing shreds of evidence showed that IL-17 was upregulated in rodent models of pathological pain in the periphery and central nervous system [22, 23]. Besides, IL-17 administration led to mechanical allodynia that could be alleviated by IL-17 antibody [24, 25]. Moreover, it has been shown that IL-17 contributed to allodynia by promoting inflammation, enhancing dorsal root ganglion (DRG) neurons' excitability, and promoting the communication of glial and spinal neurons [25-28]. Overall, these studies suggest that IL-17 plays a critical role in pathological pain and it may be an attractive therapeutic target in the management of pathological pain. Thus, in this review, we summarized the peripheral and central mechanisms of IL-17 in pathological pain.

## 2 IL-17/IL-17R IN THE NERVOUS SYSTEM UNDER PATHOLOGICAL CONDITIONS

IL-17 is involved in many human central nervous system diseases. γδ T cells producing IL-17 have been found to be present in the resected brain tissues of genetic or acquired epilepsy and their numbers are positively associated with disease severity [29]. In multiple sclerosis (MS) patients, IL-17A was significantly upregulated in the cerebrospinal fluid of MS patients and IL-17R was expressed on blood-brain barrier endothelial cells [30, 31]. This suggested that IL-17 was associated with disruption of the blood-brain barrier. Besides, IL-17A-positive lymphocytes were detected in the autoptic brain tissue of stroke patients [32].

IL-17R is upregulated in the central nervous system in a variety of central nervous system diseases. Focal cortical dysplasias can cause epilepsy by inflammatory response [33]. A study has shown that IL-17R was significantly upregulated in the cerebral cortex of focal cortical dysplasias patients and the level of IL-17R was positively associated with the frequency of seizures in focal cortical dysplasias patients [34]. In patients with mesial temporal epilepsy, it was also found that IL-17R was upregulated [35]. Experimental autoimmune encephalomyelitis (EAE) is thought that inflammatory cytokines trigger an inflammatory cascade in the central nervous system and result in myelin damage [36]. IL-17R was upregulated in the central nervous system and spinal cord of EAE mice [37]. Neuronal death is mediated by an inflammatory response after stroke [38]. It was reported that the expression of IL-17R was increased in the cortex after stroke [39]. This suggested that the interaction of IL-17 and IL-17R contributed to neuronal death after stroke. Besides, in addition to innate immune cells, the adaptive immune system is involved in Parkinson’s disease [40]. IL-17R was increased in midbrain neurons and interaction with IL-17 promoted midbrain neuron death [41]. Moreover, the upregulation of IL-17R was also thought to be associated with cognitive decline resulting from obesity [42]. There is little research on IL-17 expression in the peripheral nervous system in pathological conditions and the expression of IL-17 remains unclear in the peripheral nervous system.

## 3 PERIPHERAL MECHANISMS OF IL-17 IN PATHOLOGICAL PAIN

In the acute stage of pain, the effect of IL-17 is limited, and it may play a more important role in the late stage of pain [43]. After nerve injury, the breakdown of the blood-nerve barrier and the alteration of blood-brain barrier permeability provided a basis for T cells and IL-17 migration [44, 45]. T cells migrated to the inflamed tissue regulated by IL-17 and the connecting segment 1 (CS1) isoform of Fibronectin (FN) [46, 47]. FN-CS1 promoted mechanical allodynia rather than thermal hyperalgesia by activating the extracellular-signal-regulated kinase (ERK)/mitogen-activated protein kinase (MAPK) pathway in Schwann cells. It is reported that Schwann cells expressed IL-17RA and IL-17RB. IL-17 mediated demyelination in Schwann cells by decreasing myelin synthesis [48]. After knockout IL-17, inflammatory cell migration decreased from the periphery to the injury site and neuropathic pain was alleviated. Besides, IL-17 also promoted allodynia in pathological pain *via* interacting with macrophages in nerve injuries and enhancing the excitability of DRG neurons. A recent study has shown that IL-17 mainly co-localized with satellite glia cells in dorsal root ganglion and IL-17R was primarily co-localized with IB4-positive neurons. The interaction between IL-17 and IL-17R enhanced the frequency of action potential firings on DRG neurons and promoted the communication of glial and neurons in the dorsal root ganglion [28].

### 3.1 IL-17 and Neutrophils

Neutrophil recruitment is one of the hallmarks of pathological pain [49]. The interaction of a variety of soluble molecules and endothelial cells promotes neutrophil recruitment from the blood vessels to the inflamed tissue, such as CXC chemokines and complement component 5a (C5a) fragment [50-52]. Chemokines bind to endothelial cells through glycosaminoglycans (GAGs) in the vascular bed near the inflamed tissue, and GAGs promote the interaction of chemokines and leukocytes and enhance cell migration [50]. C5a binds to the C5a receptor expressed on neutrophils and their interactions act as chemotaxis and activate neutrophils [52]. There is a growing body of evidence that show that IL-17, a new pro-nociceptive cytokine, was involved in peripheral neutrophil recruitment in pathological pain. In a model of antigen (mBSA)-induced arthritis, the level of IL-17 in the joint lumen increased with time [53]. Intraarticularly injection of anti-IL-17 antibody with mBSA alleviated mechanical allodynia and neutrophil recruitment. After injecting Complete Freund’s adjuvant (CFA) into the plantar area, oral administration of anethole (250 mg/kg) alleviated mechanical allodynia by downregulating the levels of IL-17, TNFα, and IL-1β and inhibiting neutrophil recruitment [54]. Besides, a variety of inflammatory mediators were released after IL-17 intraarticular administration, such as TNF-a, IL-1b, CXCR1/2 chemokines ligands, MMPs, endothelins, prostaglandins, and sympathetic amines. And neutrophil recruitment is related to the interaction between TNFR1 and TNF released by resident cells [53]. Therefore, the underlying mechanism by which IL-17 promotes neutrophil recruitment and then leads to pathological pain: IL-17 promotes the release of tumor necrosis factor α (TNFα) by resident cells, and the combination of TNFα and TNF receptor 1 (TNFR1) promotes the release of proinflammatory mediators, thereby promoting neutrophil recruitment and ultimately leading to allodynia.

In addition to promoting pain through the recruitment of neutrophils, the interaction of IL-17 and neutrophil extracellular traps (NETs) also promotes hyperalgesia. NETs are large, extracellular, web-like structures and consist of cytosolic and granule proteins [55]. After neutrophil activation, NETs expand into the extracellular space. It can kill bacteria, fungi, viruses, and parasites [56-58]. IL-17 may express on NETs and triggers NETs [59, 60]. Studies have shown that infiltrating neutrophils formed NETs after spinal cord injury [61]. Due to the web-like structure of NETs, uric acid could be captured leading to local uric acid accumulation and promoting hyperalgesia during muscle trauma [62]. Besides, intraarticular injection NETs reduced the mechanical threshold in mice and joint hyperalgesia induced by NETs was prevented in mice deficient for *Tlr*4 and *Tlr*9 [63]. Moreover, eicosapentaenoic acid alleviated the hyperalgesia in oxaliplatin-induced peripheral neuropathy mice by inhibiting NETs formation and then abolishing NLR family pyrin domain containing 3 (NLRP3) inflammasome activation [64]. Therefore, NETs triggered by IL-17 may cause hyperalgesia through Toll-like receptor (TLR)-4, TLR-9 or activation of NLRP3.

### 3.2 IL-17 and Macrophages

The number of macrophages increased in the inflamed tissue, especially in chronic status [65]. Growing shreds of evidence indicated that the interaction of IL-17 and macrophages was involved in pathological pain by neuroinflammation and nociceptors activation. After nerve injury, the alteration of the blood-brain barrier and blood-nerve barrier permeability promoted peripheral IL-17 and T cells migration to damaged nerves [44, 66]. Besides, IL-23 and IL-15 as main regulators further upregulated the expression of IL-17 at the site of the damaged nerve [66]. The upregulation of IL-17 increased the expression of the macrophage marker molecule F4/80, the chemokine macrophage chemoattractant protein-1 (MCP-1), and pro-inflammatory cytokines [45, 66]. In turn, macrophages could also release IL-17 [67]. In addition, a possible mechanism for IL-17 in sexual dimorphism has been proposed that IL-23, with the help of IL-17 released by macrophages promoted female-specific mechanic hyperalgesia by activating transient receptor potential vanilloid type 1 (TRPV1)-positive nociceptors containing estrogen receptor subunit α (ERα) [67]. In contrast, IL-17 knockout didn’t reduce macrophage infiltration and mechanical allodynia in mice with chronic pelvic pain caused by severe prostatitis [68]. Therefore, further studies on the pathogenesis of IL-17 and macrophages in pathological pain are still needed.

### 3.3 IL-17 and DRG Neurons

The DRG is considered to be a relay station for sensory conduction, especially pain transmission [69]. A variety of fibers present in the axons of DRG sensory neurons convey peripheral sensory information to DRG sensory neurons [70]. C fibers are thought to play an important role in pathological pain [71]. Recently converging evidence has shown that IL-17 was involved in the nociceptive information process of DRG sensory neurons. Studies from transgenic mice provided evidence that compared with wild-type mice, IL-17 knockout mice had less mechanical allodynia in a mice model of antigen-induced arthritis (AIA) and inflammatory pain model [72, 73]. But interestingly, there was no difference in thermal hyperalgesia between wild-type mice and IL-17 knockout mice in the inflammatory pain model [72]. This suggests that the mechanism by which IL-17 mediates mechanical allodynia and thermal hyperalgesia may be different. Mechanical allodynia induced by intra-articular injection IL-17 didn’t alleviate by neutralizing TNFα or IL-6 [25]. *In vitro* experimental results showed that IL-17 contributed to the phosphorylation of protein kinase B (PKB)/Akt and ERK and the upregulation of NF-κB and transient receptor potential vanilloid 4(TRPV4) through interacting with the IL-17R of DRG sensory neurons [25, 72, 73]. Besides, IL-17 induced spontaneous discharge on DRG neurons. Selectively knock out IL-17RA on DRG neurons attenuated the frequency of action potentials firings [28]. Therefore, the underlying mechanism by which IL-17 is involved in the nociceptive information process of DRG sensory neurons could be that the interaction of IL-17 and IL-17R on DRG neurons promotes the phosphorylation of PKB/Akt and ERK, as well as the upregulation of NF-κB and TRPV4 which enhances the excitability of DRG neurons and promotes mechanical allodynia rather than thermal hyperalgesia. But these mechanisms need to be further investigated *in vivo* pathological pain models.

## 4 CENTRAL MECHANISMS OF IL-17 IN PATHOLOGICAL PAIN

It is well known that central sensitization is one of the mechanisms leading to pathological pain. The activation of spinal cord glial cells and the release of proinflammatory cytokines and chemokines promote the process of central sensitization [74, 75]. Current studies have shown that IL-17 promotes the process of pathological pain *via* activating spinal cord astrocytes and microglia.

### 4.1 IL-17 and Astrocytes

One of the mechanisms of pathological pain is the activation of astrocytes in the central nervous system. It was reported that IL-17 was upregulated in the spinal cord in pathological pain. In addition to IL-17, CCL20, a key chemokine necessary for Th17 cell migration, and JAK/STAT3, which promotes the transfer of signals from IL-17R to the nucleus, were also upregulated [76-78]. The upregulation of IL-17 was closely related to the activation of spinal astrocytes. IL-17 which originated from CD4-positive T cells promoted spinal astrocyte proliferation and activation [79]. In turn, spinal astrocytes could be activated by transient receptor potential cation (TRP) channels and kinin B1R and further produced IL-17 [80, 81]. IL-17 further interacts with spinal neurons to promote pathological pain. IL-17, which originated from spinal astrocytes, was involved in pathological pain by promoting Ca^2+^/calmodulin-dependent protein kinase II (CaMKII)-mediated c-AMP-responsive element-binding protein (CREB) phosphorylation in spinal neurons [24]. N-methyl-D-aspartate receptor (NMDAR) in spinal neurons is critical for the pathogenesis of pain [82]. IL-17 interacted with IL-17R and further promoted phosphorylating NR1 in the NMDAR of spinal neurons [83]. NR1 was an important subunit of the NMDAR to modulate NMDAR activity. In addition, IL-17 could also promote the spinal GluN2B-containing NMDAR transfer from the cytosol to the membrane surface and thus enhanced the excitatory synaptic transmission of neurons [84]. Somatostain-positive (SOM+) neurons in the spinal cord are critical for sensing mechanical pain [85]. IL-17 produced by astrocytes could enhance excitatory synaptic transmission mediated by NMDAR on SOM+ neurons and inhibit inhibitory synaptic transmission mediated by GABAR on SOM+ neurons through interaction with IL-17R on SOM+ neurons [28]. On the contrary, spinal insulin-like growth factor-1 (IGF-1), secreted by spinal astrocytes, interacted with the IGF-1 receptor on spinal neurons to inhibit the levels of spinal IL-17 and relieve chemotherapy-induced pain [86]. Interestingly, the level of IL-17 didn’t change in the trigeminal nucleus caudalis in chronic migraine mice [87]. Thus, the changes of IL-17 in the brain circuits during the pathologic pain state need to be further studied. The above evidence suggests that activated astrocytes and IL-17 play a critical role in pathological pain.

### 4.2 IL-17 and Microglia

In addition to the aforementioned spinal astrocyte, there is a close connection between spinal microglia and IL-17 in the pathological pain progress. T cells infiltrate into the spinal cord and most of them are CD3-positive CD4-positive T cells in pathological pain. Intrathecal administration of human umbilical cord-derived mesenchymal stem cells (HUC-MSCs) or oral administration of Crotalphine (CRO) alleviated neuropathic pain or pain induced by experimental autoimmune encephalomyelitis through inhibiting glial cells activation and IL-17 release [22, 23]. But these studies didn’t clarify how IL-17 and spinal microglia interact in the pain progress. Huo *et al*. further proved that IL-17R mainly co-localized with microglia marker [27]. Intrathecal administration of IL-17 antibody inhibited microglia activation and alleviated bone cancer pain. Studies from transgenic mice provided further evidence that IL-17 knockout inhibits spinal glial cell activation and alleviated mechanical allodynia in the neuropathic pain model [88]. Therefore, the above studies indicate that IL-17 originated from T cells, interacts with IL-17R on spinal microglia, and activates microglia, ultimately causing pain. However, the signal transduction in microglia induced by the interaction between IL-17 and IL-17R remains to be further studied.

## 5 DRUGS TARGETING IL-17 AND TH17 CELLS

The methods of targeting IL-17 include inhibition of Th17 differentiation by IL-23 inhibitors, inhibition of IL-17, and inhibition of IL-17R (Table **1**). First of all, the interaction of IL-23 and IL-23R promotes the differentiation of Th17 cells and thus may inhibit IL-17 by inhibiting IL-23. Tildrakizumab, a specific antibody to IL-23p9, has been tested in patients with plaque psoriasis. A Phase III trial showed that compared with 6% in the placebo group, 62% of patients receiving a 200 mg dose achieved Psoriasis Area and Severity Index (PASI) 75 at week 12 and 64% of patients receiving 100 mg dose achieved PASI 75 at week 12 [89]. Secondly, some antibodies act directly on IL-17. Secukinumab (AIN457), a human monoclonal IgG1-kappa antibody, inhibits IL-17 by binding IL-17. It is approved by the US FDA for the treatment of plaque psoriasis, psoriatic arthritis, and ankylosing spondylitis. A Phase III trial showed that compared with 19.6% in the placebo group, the American College of Rheumatology 20% improvement criteria (ACR20) response rate at week 24 was 35.2% in the secukinumab group [90]. Ixekizumab (LY2439821), a recombinant, high affinity, humanized monoclonal IgG4-kappa antibody, binds and inhibits IL-17. It is approved by the US FDA for the treatment of plaque psoriasis and psoriatic arthritis. The results of a Phase II trial have been published. Compared with the placebo group, ACR20 responses at week 12 were better in patients with an inadequate response to TNF inhibitors [91]. Bimekizumab is a novel monoclonal antibody targeting IL-17A and IL-17F. A Phase II trial with bimekizumab in patients with psoriatic arthritis showed that bimekizumab treatment was associated with long-term sustained improvements in pain and fatigue, reducing the overall impact of psoriatic arthritis on patients [92]. Brodalumab (AMG827), a fully human IgG2 monoclonal antibody, binds to the IL-17 receptor. It is approved by the US FDA for the treatment of moderate-to-severe plaque psoriasis. A Phase II trial with brodalumab in patients with rheumatoid arthritis (RA) showed that after brodalumab treatment, there is no meaningful clinical efficacy in patients with RA [93].

## CONCLUSION

**Fig. (1):**
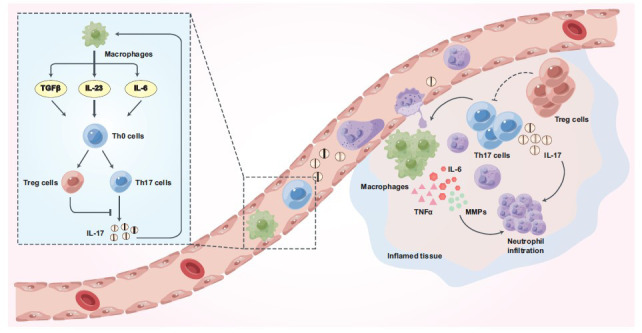
The peripheral mechanisms of IL-17 in pathological pain. Under pathological pain conditions, Th0 cells differentiate into Th17 cells under the action of IL-23, and Th17 cells enter the damaged tissues to secrete IL-17 with the help of chemokines. Besides, macrophages infiltrate the damaged tissues and release a series of cytokines under the action of IL-17, including IL-6, TNFα, and MMPs. These cytokines and IL-17 promote neutrophil recruitment and infiltration and further promote pathological pain.

**Fig. (2):**
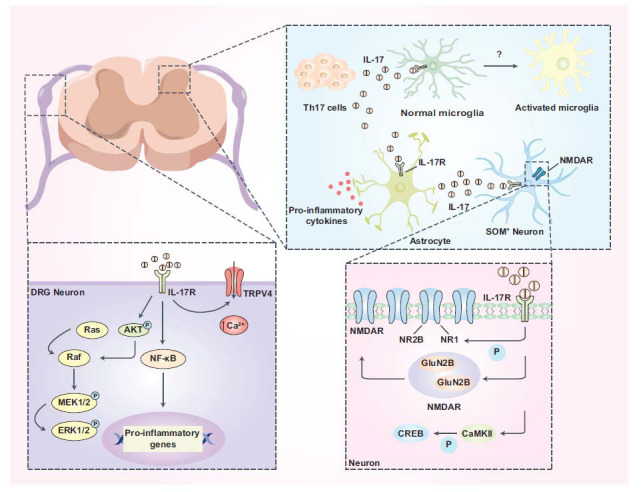
The spinal cord and DRG mechanisms of IL-17 in pathological pain. Under pathological pain, the breakdown of the blood-nerve barrier promotes that Th17 cells infiltrate the spinal cord and release IL-17. IL-17 activates microglia by binding to IL-17R. Besides, IL-17 also activates astrocytes in the same way and astrocytes release pro-inflammatory cytokines and IL-17. IL-17 released by astrocytes promotes the communication of SOM+ neurons and astrocytes by binding to IL-17R on SOM+ neurons. Moreover, IL-17 promotes the NR1 subunit phosphorylation of NMDAR on neurons and NMDAR migration from the cytoplasm to the membrane and the CREB phosphorylation of CaMKII-mediated. On DRG neurons, IL-17 interacts with IL-17R triggering a cascade of intracellular reactions that promote the transcription of proinflammatory genes thus promoting DRG neurons' excitability.

In this review, we summarized the upregulation of IL-17 in the peripheral and central nervous systems of rodent models of pathological pain (Table **2**). These studies indicated that the upregulation of IL-17 promoted the development of pathological pain by promoting neuroinflammation, enhancing the excitability of DRG neurons, and promoting the communication of glial and spinal neurons (Figs. **1** and **2**). However, these studies raise other questions.

Firstly, the current studies of pathological pain models only focus on IL-17 in the periphery and spinal cord. Several brain circuits are known to be involved in the development of pathological pain [94, 95]. However, there was a study that suggested that the level of IL-17 didn’t change in the trigeminal nucleus caudalis in chronic migraine mice [87]. This contradicts the results of the spinal cord and peripheral studies. Thus, it is necessary to determine whether IL-17 is involved in the processing of pathological pain by brain circuits.

Secondly, most of the current studies that illustrated the role of IL-17 in pathological pain have used male rodents. Only one study investigated the mechanisms of IL-17-mediated female neuropathic pain. Several lines of evidence show that clinical pain experience is different between men and women [96, 97]. Moreover, ample epidemiologic evidence shows that chronic pain is more common in women [98, 99]. Multiple mechanisms are thought to be involved in this process, such as the effects of sex hormones, differences in endogenous opioid function, and cognitive/affective influences [98, 99]. Thus, the mechanisms of IL-17 in pathological pain need to be further investigated.

Moreover, the mechanism by which IL-17 enhances DRG neurons' excitability has only been demonstrated *in vitro* studies, but whether IL-17 causes hyperalgesia in rodents by enhancing the excitability of DRG neurons still needs further study.

Besides, the current studies indicate that peripheral IL-17 may not be associated with thermal hyperalgesia, but inhibiting the level of spinal cord IL-17 can alleviate thermal hyperalgesia. Thus, the underlying mechanisms need to be further studied.

In current clinical trials, antibodies targeting IL-17 are used in humans either intravenously or subcutaneously (Table **1**). The use of live attenuated varicella vaccine as a possible adjunct therapy in the treatment of psoriasis could play a therapeutic role by regulating Th17/Treg balance [100]. Besides, autologous haematopoietic stem cell transplantation was critical for MS patients by inhibiting Th17 cytokines [101]. RA patients who had an inadequate response to conventional treatments were treated with Guluronic Acid, a new nonsteroidal anti-inflammatory drug, and the expression of the RORct gene was reduced [102]. Montelukast decreased the expression of IL-17 and may serve as a potential adjuvant therapy for patients with RA [103]. IL-23 is critical for Th17 maintenance. Ustekinumab significantly decreased IL-17 by inhibiting IL-23p40 in patients who received peripheral blood-mobilized hematopoietic cell transplantation [104].

Finally, current research showed that IL-17A is the most intensively investigated in pathological pain. There is a clinical study showing that inhibiting IL-17F could alleviate pain [92]. Thus, more research on other IL-17 family members should follow. In addition to IL-17A, other IL-17 family members also promote neutrophil migration. Research showed that intraperitoneal injection of IL-17B caused significant neutrophil migration [105]. Besides, IL-17A, IL-17C, and IL-17F promoted neutrophil-mediated immunity by inducing inflammatory cascades [106]. IL-17E, a new TH2 cytokine, promoted airway eosinophilia in mice, as well as promoted neutrophil migration [107]. Like IL-17A, other IL-17 family members also interact with macrophages. Macrophages could synthesize IL-17A and IL-17F, and in turn, IL-17A and IL-17F promoted lung cancer cell growth by macrophages [108, 109]. Moreover, macrophages expressed IL-17E receptors and responded to IL-17E [110]. IL-17E neutralization reduced macrophage infiltration [111]. In addition to IL-17A, there are few studies on astrocytes and other IL-17 family members. Knockout ACT1 (a key transcription factor for signals mediated by IL-17A, IL-17F, and IL-17C) reduced the number of infiltrating inflammatory cells and ameliorates experimental autoimmune encephalomyelitis [112]. Studies between the IL-17 family and DRG neurons or microglia currently focus on IL-17A and the interaction between other IL-17 family members and DRG neurons or microglia should be further investigated.

Overall, these studies indicate that IL-17 is an attractive target in pathological pain treatment, but the underlying mechanisms still need to be investigated.

## Figures and Tables

**Table 1:** Available approaches targeting IL-17 and IL-17R.

**Drug**	**Target**	**Disease**	**Phase**	**Treatment**	**Status**	**PMID or ** **Identifier**
Ixekizumab (LY2439821)	IL-17A	RA	II	LY2439821 (3, 10, 30, 80, 180 mg, s.c.) was administrated	Completed	NCT00966875
		Psoriasis	III	LY2439821 (160 mg, s.c.) was given at week 0 and then LY2439821 (80 mg, s.c.) was given again at week 2 or week 4	Completed	NCT02387801
Secukinumab (AIN457)	IL-17A	RA	III	AIN457 (10 mg/kg, i.v.) was given at baseline, and then AIN457 (75, 150 mg/kg, s.c.) was administrated every 4 weeks starting at week 8	Completed	NCT01377012
		Psoriasis	III	Secukinumab (150, 300 mg, s.c.) was given on day 0, and at weeks 1, 2, 3, 4, and then every 4 weeks	Completed	NCT01961609
		Ankylosing spondylitis	III	Secukinumab (6 mg/kg, i.v.) was given in 1.0 mL pre-filled syringes	Completed	NCT02750592
		Psoriatic arthritis	III	Secukinumab (6 mg/kg, i.v.) was given at baseline followed by secukinumab (3 mg/kg i.v.) every 4 weeks starting at week 4 through week 48	Completed	NCT04209205
Brodalumab (AMG827)	IL-17RA	RA	II	Brodamulab (70, 140, 210 mg, s.c.) was given on day 1 and weeks 1, 2, 4, 6, 8, and 10	Completed	NCT00950989
		Psoriasis	VI	Brodamulab (210 mg, s.c.) was given using prefilled syringes	Completed	NCT03403036
Bimekizumab	IL-17A and IL-17F	Moderate to severe plaque psoriasis	III	Bimekizumab was administrated through subcutaneous injection during the treatment period	Completed	NCT05020249
		Ankylosing spondylitis	III	Bimekizumab was given at pre-specified time points	Completed	NCT03928743
		Psoriatic arthritis	II	Bimekizumab was given up to 2 years	-	NCT03347110
Tildrakizumab (SCH-900222)	IL-23p19	Plaque psoriasis	III	Tildrakizumab (100, 200 mg, s.c.) were given	Completed	NCT01729754
miR-21	IL-17	RA	-	MiR-21 levels significantly decreased in RA patients	-	25164131
miR-146a	IL-17	RA	-	MiR-146a intensely expressed in synovium with high expression of IL-17 in RA patients	-	20840794
miR-23b	IL-17	RA	-	MiR-23b downregulated in the synovial tissues of rheumatoid arthritis patients and the kidney tissues of SLE patients and IL-17 suppressed the expression of miR-23b	-	22660635
lncRNA CASC2	IL-17	RA	-	lncRNA CASC2 was downregulated in RA and the overexpression of lncRNA CASC2 inhibited IL-17 expression	-	32186765
lncRNA OIP5-AS1	IL-17	UC	-	Vitamin D treatment could decrease IL-17 and suppress Th17 polarization by regulating the lncRNA OIP5-AS1 levels in UC	-	35767888
lncRNA XIST	IL-17	Psoriasis	-	Serum lncRNA XIST was increased in patients with psoriasis and XIST silencing suppressed the discharge of IL-17	-	35231918
lncRNA STAT4-AS1	Th17 differentiation	Asthma	-	STAT4-AS1 inhibits the mutual binding of RORγt and IL-17 gene promoter and eventually inhibits Th17 differentiation	-	35528611
lncRNA HOTAIR	Th17 differentiation	Hepatic fibrosis	-	Arsenite promotes RORγt-mediated Th17 cell differentiation through HOTAIR	-	36332283

**Abbreviations:** EAE: experimental autoimmune encephalomyelitis; i.v.: intravenously; RA: rheumatoid arthritis; s.c.: subcutaneously; SLE: systemic lupus erythematosus; UC: ulcerative colitis.

**Table 2:** Summary of therapeutic potential of IL-17 in pathological pain.

**Model**	**Treatment**	**Effects**	**Mechanisms**	**References**
SNL-induced neuropathic pain rats	-	MPWT↓	Astrocytes proliferation and activation↑ IL-1β, IL-6↑	[92]
EAE-induced neuropathic pain mice	Selective B_1_R antagonist (DALBK, 50 nmol/kg, i.p., twice per day) was administrated from day 0 to day 5 after EAE induction EAE model was established in B_1_R knockout mice	Tactile hypersensitivity↓ PWL↑	IL-17, IFN-γ, IL-6, CXCL-1/KC, COX-2, NOS2↓ Glial activation↓	[13]
SCI-induced neuropathic pain rats	Spinal cord injury model was established in rats	BBB scores↓	IL-6, IL-17, IL-21, IL-23↑ p-STAT3↑	[112]
SCI-induced neuropathic pain rats	IL-17 inhibitor (25mg/kg/d, i.p.) was administrated 30 minutes after spinal cord injury for 14 consecutive days	BBB scores↑	IL-1β, IL-6, TNF-α, IL-17↓ JAK2, STAT1, STAT3↓ GFAP, VEGF↓	[107]
SCI-induced neuropathic pain rats	CCL20 antibody (100 μg/kg, i.p.) was administrated after spinal cord injury for 28 consecutive days	BBB scores↑ Spinal water content↓	IL-1β, IL-6, TNF-α, IL-17↓ Th17 cells recruitment↓	[33]
IS-induced chronic migraine mice	-	Mechanical threshold↓	IL-17-	[108]
SNI-induced neuropathic pain mice	IL-17 (5, 25 ng/mice, intraplantar injection; 10, 25, 50 ng/mice, intraneural injection; 25, 50 ng/mice, i.t.) was administrated before behavior tests	MPWT↓ PWL↓	Glial cells activation↑ T cells and macrophages recruitment↑	[39]
Cancer-induced pain mice	IL-17/IL-17A antibodies (20 μg/mouse, i.t.) was administrated on day 14 after model establishment	MPWT↑ NSF↓	Microglia activation↓	[34]
Cancer-induced pain rats	Liquiritin (20, 100, 500, 1000 μg/kg, i.t.) was administrated for 7 days before behavior tests	MPWT↑	Astrocytes activation↓ IL-17, IL-1β↓ CXCL1/CXCR2 signaling pathway↓	[64]
SNL-induced neuropathic pain rats	HUC-MSCs (20 μl/rat, i.t.) was administrated on day 3 after SNL	MPWT↑ PWL↑	Astrocytes and microglial activation↓ IL-17, IL-1β↓	[8]
SNL-induced neuropathic pain mice	IL-17A antibody (2 μg/mice, i.t.) was administrated on day 7 after SNL	MPWT↑ PWL↑	CaMKII/CREB signaling pathway activation↓	[105]
CCI-induced neuropathic pain rats	CS1 (50 μg/ml in 5 μl PBS) was immediately administrated to injury nerve after CCI	MPWT↑ PWL-	IL-17A↓ ERK/MAPK↓	[50]
Chemotherapy-induced neuropathic pain mice	IL-17A (100 ng, i.pl.) was administrated to ERα conditional knockout mice	MPWT↑	Estrogen receptor subunit α (ERα) in TRPV1+ nociceptors↓	[53]
CCI-induced neuropathic pain mice	CCI model was established in RAG-1 knockout mice	PWL↑	IL-17A↓ MCP-1↓ Macrophage↓	[40]
AIA-induced neuropathic pain mice	IL-17 antibody (100 μg, i.p.) was administrated daily for 3 days before AIA induction	MPWT↑	p-PKB/Akt↓ p-ERK1/2↓ DRG sensory neurons excitability↓	[77]
AIA-induced neuropathic pain mice	AIA-induced neuropathic pain model was established in IL-17A knockout mice	MPWT↑ PWL↑	Sensory nociceptive neurons sensitization↓	[14]
SNL-induced neuropathic pain mice	Sciatic nerve ligation model was established in T lymphocyte-deficient nude mice	-	IL-17↓ IL-17-positive cells↓ Macrophages recruitment↓	[45]
SNI-induced neuropathic pain mice	Sciatic nerve ligation model was established in IL-17-/-mice	MPWT↑ PWL↑	Astrocytes proliferation↓ proinflammatory cytokines secretion↓	[11]
Zymosan-induced inflammatory pain mice	Zymosan-induced inflammatory pain model was established in IL-17A-deficient (IL-17A^−/−^) mice	MPWT↑ PWL↓	TRPV4↓ ERK, NF-κB↓	[85]
CFA-induced inflammatory pain mice	Anethole (250 mg/kg, i.g.) was administrated daily for 7 consecutive days after CFA injection	MPWT↑	MPO activity↓ TNF-α, IL-17, IL-1β↓	[78]
Model of antigen (mBSA)-induced articular pain mice	Anti-IL-17 antibody (2.25 μg, i.a.) was administered simultaneously with mBSA TNFR1^-/-^ mice were injected with mBSA Infliximab (10 mg/kg, i.p. 48 h and 60 min before IL-17 injection), anti-TNF-α antibody was administrated DF-2156 (30 mg/kg, i.v.), was administrated IL-1R antibody (50 mg/kg, i.v. 30 min before and 3.5 h after stimuli injection) was administrated Bosentan (100 mg/kg, p.o. 60 min before IL-17 injection) was administrated Indomethacin (5 mg/kg, i.p. 30 min before stimuli injection) or guanethidine (30 mg/kg, s.c. 60 min before stimuli injection) was administrated	MPWT↑	Neutrophil migration↓ TNF-a, IL-1β, CXCR1/2 chemokines ligands, MMPs, endothelins, prostaglandins and sympathetic amines↓	[72]
NTG-induced chronic migraine rats	NTG (10 mg/kg, s.c.) was administrated five times over two days	MPWT↓	IL-17A, IL-1β, IL-6, TNF-α↑	[9]
Cancer-induced pain rats	LTTL gel (0.5 g/cm^2^/d) was administrated to the skin for 21 days after a day of model establishment	MPWT↑ PWL↑	TRP channels in DRG↓ IL-17A↓	[101]
PAg-induced inflammatory pain mice	Experimental autoimmune prostatitis models were established in IL-17 knockout mice	Response frequency	-	[62]

**Abbreviations:** AIA: antigen-induced arthritis; BBB: Basso, Beattie, and Bresnahan; CaMKII: Ca2+/calmodulin-dependent protein kinase II; CCI: chronic constriction injury; CFA: Complete Freund’s adjuvant; CREB: c-AMP-responsive element-binding protein; CS1: connecting segment 1; DRG: dorsal root ganglion; ERK: extracellular-regulated kinase; ERα: estrogen receptor subunit α; HUC-MSCs: human umbilical cord-derived mesenchymal stem cells; i.a.: intraarticularly; i.p.: intraperitoneally; i.v.: intravenously; IL-17: interleukin-17; IL-1β: interleukin-1β; IS: inflammatory soup; LTTL: Long-Teng-Tong-Luo; MAPK: mitogen-activated protein kinase; MCP-1: macrophage chemoattractant protein-1; MMPs: matrix metalloproteinases; MPO: myeloperoxidase; MPWT: mechanical paw withdrawal threshold; NF-κB: nuclear factor κB; NSF: number of spontaneous flinches; NTG: nitroglycerin; p.o.: orally; PAg: prostate antigen; PKB: protein kinase B; PWL: paw withdrawal latency; RAG-1: recombination-activating gene-1; s.c.: subcutaneously; SCI: spinal cord injury; SNL: sciatic nerve ligation; TNF: tumor necrosis factor; TNFR1: tumor necrosis factor receptor 1; TRP channels: transient receptor potential cation channels; TRPV4: transient receptor potential vanilloid 4; -: no change; ↓: downregulated; ↑: upregulated.

## LIST OF ABBREVIATIONS

AIA: Antigen-induced Arthritis
C/EBPs: CCAAT/Enhancer Binding Proteins
C5a: Complement Component 5a
CaMKII: Ca^2+^/Calmodulin-dependent Protein Kinase II
CFA: Complete Freund’s Adjuvant
CREB: c-AMP-responsive Element-binding Protein
CRO: Crotalphine
CS1: Connecting Segment 1
DRG: Dorsal Root Ganglion
EAE: Experimental Autoimmune Encephalomyelitis
ERK: Extracellular-signal-regulated Kinase
ERα: Estrogen Receptor Subunit α
FN: Fibronectin
GAGs: Glycosaminoglycans
HUC-MSCs: Human Umbilical Cord-derived Mesenchymal Stem Cells
IL-17: Interleukin-17
IL-17R: IL-17 Receptor
MAPK: Mitogen-activated Protein Kinase
mBSA: Model of Antigen
MCP-1: Macrophage Chemoattractant Protein-1
MS: Multiple Sclerosis
NETs: Neutrophil Extracellular Traps
NF-κB: Nuclear Factor-κB
NK cells: Natural Killer Cells
NLRP3: NLR Family Pyrin Domain Containing 3
NMDAR: N-methyl-D-aspartate Receptor
PKB: Phosphorylation of Protein Kinase B
RA: Rheumatoid Arthritis
RIH: Remifentanil-induced Hyperalgesia
TGF-β: Transforming Growth Factor β
Th17 cells: T Helper 17 Cells
TLR4: Toll-like Receptor-4
TNFR1: TNF Receptor 1
TRP channels: Transient Receptor Potential Cation Channels
TRPV1: Transient Receptor Potential Vanilloid Type 1
TRPV4: Transient Receptor Potential Vanilloid Type 4
